# Overexpression of Snail induces epithelial–mesenchymal transition and a cancer stem cell–like phenotype in human colorectal cancer cells

**DOI:** 10.1002/cam4.4

**Published:** 2012-06-08

**Authors:** Fan Fan, Shaija Samuel, Kurt W Evans, Jia Lu, Ling Xia, Yunfei Zhou, Eric Sceusi, Federico Tozzi, Xiang-Cang Ye, Sendurai A Mani, Lee M Ellis

**Affiliations:** 1 Department of Cancer Biology, The University of Texas MD Anderson Cancer CenterHouston, Texas; 2 Department of Molecular Pathology, The University of Texas MD Anderson Cancer CenterHouston, Texas; 3 Department of Surgical Oncology, The University of Texas MD Anderson Cancer CenterHouston, Texas; 4 The University of Texas Graduate School of Biomedical SciencesHouston, Texas; 5 Metastasis Research Center, The University of Texas MD Anderson Cancer CenterHouston, Texas

**Keywords:** Cancer stem cells, colorectal cancer, EMT, migration, Snail

## Abstract

Epithelial–mesenchymal transition (EMT) is a critical process providing tumor cells with the ability to migrate and escape from the primary tumor and metastasize to distant sites. Recently, EMT was shown to be associated with the cancer stem cell (CSC) phenotype in breast cancer. Snail is a transcription factor that mediates EMT in a number of tumor types, including colorectal cancer (CRC). Our study was done to determine the role of Snail in mediating EMT and CSC function in CRC. Human CRC specimens were stained for Snail expression, and human CRC cell lines were transduced with a retroviral Snail construct or vector control. Cell proliferation and chemosensitivity to oxaliplatin of the infected cells were determined by the MTT (colorimetric 3-(4,5-dimethylthiazol-2-yl)-2,5-diphenyltetrazolium bromide) assay. Migration and invasion were determined in vitro using modified Boyden chamber assays. EMT and putative CSC markers were analyzed using Western blotting. Intravenous injection of tumor cells was done to evaluate their metastatic potential in mice. Snail was overexpressed in human CRC surgical specimens. This overexpression induced EMT and a CSC-like phenotype in human CRC cells and enhanced cell migration and invasion (*P* < 0.002 vs. control). Snail overexpression also led to an increase in metastasis formation in vivo (*P* < 0.002 vs. control). Furthermore, the Snail-overexpressing CRC cells were more chemoresistant to oxaliplatin than control cells. Increased Snail expression induces EMT and the CSC-like phenotype in CRC cells, which enhance cancer cell invasion and chemoresistance. Thus, Snail is a potential therapeutic target in metastatic CRC.

## Introduction

Epithelial–mesenchymal transition (EMT) – which is characteristic of embryonic development, tissue remodeling, and wound healing – is a crucial process in tumor progression that causes epithelial cells to acquire fibroblast-like properties and show reduced intercellular adhesion and increased motility [1]. Several transcription factors have been implicated in the control of EMT, including Snail, Slug, Twist, ZEB1/2, SIP1, and E12/E47 [2–5]. Snail, a member of the Snail family of zinc finger transcription factors, is a central mediator of EMT and induces EMT, in part, by directly repressing epithelial markers such as E-cadherin (a gatekeeper of the epithelial phenotype) and by upregulating mesenchymal markers. Snail plays a role in the pathogenesis of several malignant neoplasms, predominantly by enhancing invasiveness and metastatic behavior. In a transgenic mouse model of breast cancer, Snail expression was associated with a more aggressive phenotype and poorer survival rates [6]. Similarly, in hepatocellular carcinoma and breast cancer, increased Snail expression was associated with increased rates of distant metastases and poorer clinical outcome [7, 8]. Furthermore, patients with Snail-expressing colon and ovarian carcinoma tumors were found to have a higher risk for distant metastases than other patients [9, 10].

Several recent reports have suggested that EMT also results in the acquisition of other properties involved in carcinoma progression, such as increased resistance to apoptosis and the acquisition of stem cell–like properties. These properties have been observed in epithelial cells ectopically overexpressing Snail. For instance, cells that have undergone Snail-induced EMT present a CD44^high^/CD24^low^ cell surface protein signature, which is similar to that previously identified for cancer stem cells (CSCs; [11]). Breast cancer cells overexpressing Snail can form mammospheres and differentiate into various lineages of cells including myoepithelial and luminal epithelial cells. Snail is also required for the expression of putative markers of stem cells, such as aldehyde dehydrogenase 1 (ALDH1; [12]), and can induce the expression of stemness-promoting genes, such as Nanog, KLF4, and TCF-4 [13]. Because of the acquisition of these properties, Snail has been associated with higher rates of recurrence in a murine model of breast cancer [6]. Therefore, elevated Snail expression in epithelial cancer cells may promote the CSC-like phenotype and subsequently facilitate the dissemination of epithelial tumors.

Analysis of Snail in human colorectal cancer (CRC) specimens in a prior study showed that 78% of the tumor samples examined overexpressed this protein [9]. Upregulation of Snail and the associated transcriptional repression of E-cadherin may play a role in the progression of CRC [14, 15]. The specific role of Snail in CRC has not been fully elucidated. Our study demonstrated that upregulation of Snail in CRC cells confers increased cell motility and invasiveness in vitro and increased metastatic potential in vivo. In addition, we showed that high levels of Snail expression in CRC cells are associated with increased chemoresistance.

## Materials and Methods

### Reagents and antibodies

Antibodies used for flow cytometry were phycoerythrin-conjugated anti-CD133 and phycoerythrin-conjugated mouse-IgG1 (Miltenyi Biotec, Auburn, CA) and fluorescein isothiocyanate (FITC)-conjugated anti-CD44 and FITC-conjugated mouse-IgG2b (BD Biosciences, San Jose, CA). Antibodies used for immunohistochemical analysis or Western blotting were rat anti-Snail, rabbit anti-CD133, rabbit anti-caspase-3 and anti-cleaved caspase-3, rabbit anti-PARP and anti-cleaved PARP (Cell Signaling Technology, Danvers, MA), mouse anti-CD44 (Abcam), mouse anti-E-cadherin (BD Biosciences), and mouse anti-fibronectin (Millipore, Billerica, MA).

### Human tissue specimens and cell lines

Formalin-fixed, paraffin-embedded primary and metastatic CRC surgical specimens were obtained from an established tumor bank at The University of Texas MD Anderson Cancer Center following protocols approved by the institutional review board.

### Cell lines and culture conditions

The human colon cancer cell lines HCT-116, RKO, SW480, SW620, HT29, SW48, and LS174T were obtained from the American Type Culture Collection. KM12L4 cell line was obtained from Isaiah J. Fidler, D.V.M., Ph.D. (The University of Texas MD Anderson Cancer Center). GEO cell line was obtained from Douglas D. Boyd, Ph.D. (The University of Texas MD Anderson Cancer Center). Cell lines were maintained in minimal essential medium (MEM) supplemented with 10% fetal bovine serum (FBS), penicillin–streptomycin, vitamins, sodium pyruvate, l-glutamine, and nonessential amino acids (Life Technologies, Grandisland, NY) at 37°C in 5% CO_2_. Cells were confirmed to be free of mycoplasma using the “MycoAlert” mycoplasma detection kit (Lonza Group, Allendale, NJ). Results from all in vitro studies were confirmed in at least three independent experiments. The Snail-overexpressing cell lines (HT29/Snail and HCT116/Snail) and their respective control cell lines were transduced with either pBabe-puro encoding human Snail or empty pBabe vector, respectively, and then selected by culturing in 2 μg/mL puromycin (Sigma-Aldrich) for 10 days, cells were then maintained in 1 μg/mL of puromycin.

### Immunohistochemistry

Twenty human paraffin-embedded, formalin-fixed tumor tissues (10 primary and 10 metastatic [not matched]) were sectioned into 5 μm thick slices. Sections were deparaffinized in xylene and rehydrated in a series of graded ethanols, and their antigens were retrieved by heating the slides in a citrate buffer (pH 6.0). Endogenous peroxidase activity was blocked with 3% H_2_O_2_ in methanol for 12 min at room temperature. The sections were then incubated for 1 h at room temperature with 4% gelatin protein blocking buffer. This procedure was followed by incubation with the primary antibodies at 1:500 dilution overnight at 4°C. Negative controls were obtained by replacing the primary antibodies with control isotype IgG. We used MACH 4HRP polymer (Biocare Medical, Concord, CA) as a secondary antibody and incubated our slides with it at room temperature for 30 min. Color was developed with 3,3′-diaminobenzidine and sections were counterstained with hematoxylin (Sigma-Aldrich, St. Louis, MO).

### Proliferation and chemosensitivity assays

Rates of proliferation and sensitivity to drugs were assessed using the colorimetric 3-(4,5-dimethylthiazol-2-yl)-2,5-diphenyltetrazolium bromide (MTT) assay (Sigma-Aldrich) as described previously [16] with minor modifications. Cells (2 × 10^3^) of each cell line were plated in 100 μL of medium per well in 96-well plates; the next day, the medium was changed to 1% FBS MEM; and cells were treated for 72 h with or without the addition of oxaliplatin (0.2, 2, and 20 μmol/L). After the incubation, the MTT assay was performed.

### Flow cytometry analysis

Cells were prepared for analysis of cell surface marker expression by plating cells at 60% confluence the day before analysis. After trypsinizing the cell, cells were washed in phosphate buffered saline and resuspended in 1% bovine serum albumin plus fluorophore-conjugated primary antibodies and incubated for 30 min at room temperature. Samples were then washed and analyzed using a Cell Lab Quanta flow cytometer (Beckman Coulter, Nyon, Switzerland), and data analysis was done using FlowJo software (Tree Star, Inc., Ashland, OR).

### Sphere-forming assay

The ability of cell lines to form spheres in suspension was evaluated with a modified method [17]. The stem cell medium consisted of Dulbecco's modified Eagle's medium (DMEM)/F12 supplemented with 1× B27 (Life Technologies) 20 μg/mL epidermal growth factor, and 20 μg/mL fibroblast growth factor-basic (Invitrogen) and penicillin–streptomycin. A single cell was sorted into each well of a 96-well ultra-low-attachment plate (Corning Life Sciences, Tewksbury, MA) containing 200 μL of stem cell medium per well. After 7 days of incubation, the total number of spheres greater than 50 μm in diameter per well was quantified by counting under light microscopy.

### Migration and invasion assays

Migration assays were conducted as described previously [18] with minor modifications. Equal numbers (1.25 × 10^5^ cells/well) of control or Snail-expressing cells were suspended in 0.5 mL of 1% FBS MEM and placed in the top chamber of the well; 0.750 mL of 10% FBS MEM was added to the bottom compartment. Following a 48-h incubation, nonmigrating cells were scraped from the membrane of the top compartment, and cells that had migrated through the membrane were fixed and stained using the Protocol Diff-Quik 3 stain set (Siemens Healthcare Diagnostics). Membranes were excised and mounted on a standard microscope slide (Matheson Scientific). The numbers of cells that migrated were determined from five random high-power fields (HPFs) visualized at 200× magnification.

The invasion assays used identical methods, except that the cells were placed in the top compartment of a modified Boyden chamber with a Matrigel-coated membrane. The numbers of invading cells were quantified from five random HPFs visualized at 200× magnification.

### Western blotting

Western blotting was conducted as described previously [16] with minor modifications using antibodies as described above. Vinculin and β-actin served as a protein loading controls.

### Annexin V-FITC staining

Apoptosis was determined using a FITC-conjugated Annexin V (Annexin V-FITC)/propidium iodide apoptosis detection kit (BD Biosciences) according to the manufacturer's instructions. Cells (5 × 10^5^) were resuspended in 100 μL of 1× binding buffer, 5 μL of Annexin V-FITC, and 5 μL of propidium iodide. After a 15-min incubation at room temperature in the dark, 400 μL of 1× binding buffer was added, and the cells positive for Annexin V-FITC and/or propidium iodide were analyzed using a BD FACS flow cytometer (BD Biosciences).

### Snail knockdown by siRNA

Cells (5 × 10^4^) were seeded in 12-well plates. When the cell density reached ∼50% confluence, the cells were transfected with either 20 nmol/L control siRNA or with a Snail-specific siRNA (Dharmacon RNAi Technologies, Lafayette, CO). Transfections were carried out using the X-tremeGENE siRNA transfection reagent (Roche, Indianapolis, IN) according to the manufacturer's instructions. The next day, the medium was changed to 1% FBS MEM with or without oxaliplatin, and cells were exposed to the treatment for 72 h. An MTT assay was performed to quantify the effect of Snail knockdown on chemosensitivity.

### In vivo metastasis assay

To evaluate the metastatic potential of Snail-overexpressing cells, we stably infected HT29/Control and HT29/Snail cells with a luciferase reporter gene lentiviral construct. Luciferase-labeled cells (1.5 × 10^6^) were suspended in 100 μL of Hank's buffered salt solution and injected intravenously via the tail vein. The incidence and volume of metastases were estimated by serial imaging of mice for bioluminescence using the IVIS 100 imaging system coupled to a data-acquisition personal computer equipped with Living Image software (Xenogen, Baltimore, MD). The mice were anesthetized with a 1.5% isoflurane-oxygen mixture and injected intraperitoneally with luciferase potassium salt solution (Sigma-Aldrich) at a dose of 150 mg/kg body weight and imaged 10 min later. The photon emission level (representative of luciferase activity) was used assess the relative tumor burden in the mice. Mice were sacrificed when three mice in any group became lethargic. All animal studies were conducted under approved guidelines of the Animal Care and Use Committee of the MD Anderson Cancer Center.

### Statistical analyses

Densitometry for Western blots were analyzed by using Image J software (NIH Image). For the in vitro studies, statistical analyses were done using Student's *t*-test (Microsoft office Excel 2007, Redmond, WA). For the in vivo studies, the chi-square test was used to determine differences in tumor incidence, and differences in the mean number of metastases were determined using the Student's *t*-test (Microsoft office Excel 2007). All statistical tests were two-sided, and *P* values <0.05 were considered to be statistically significant.

## Results

### Snail is expressed in human CRC surgical specimens and human CRC cell lines

**Figure 1:**
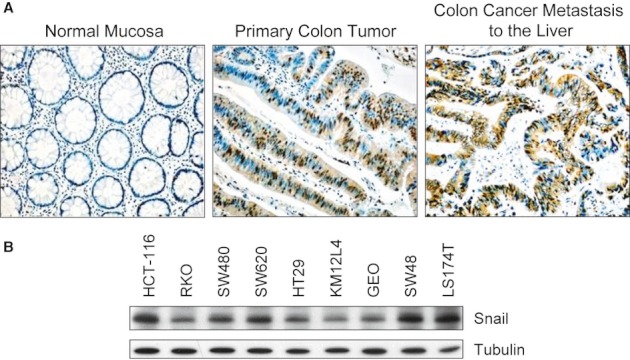
Snail expression in human CRC surgical specimens and human CRC cell lines. (A) Snail protein expression was assessed in human primary and metastatic CRC specimens by immunohistochemical analysis. Representative images are shown. (B) Western blotting showed that all the studied CRC cell lines expressed Snail.

Pena et al. reported that Snail was expressed in 18 (56.3%) of 32 tumor samples but normal tissues did not demonstrate Snail expression [19]. To briefly validate their results, immunohistochemistry was performed to evaluate the expression of Snail in 10 primary CRC surgical specimens and 10 liver metastases. Snail-positive cells were identified in the tumor epithelium, with minimal Snail expression in normal mucosa (Fig. 1A). All the CRC cell lines studied expressed Snail in Western blot analysis (Fig. 1B).

### Snail induces EMT and increases cell migration and invasion of CRC cells

**Figure 2:**
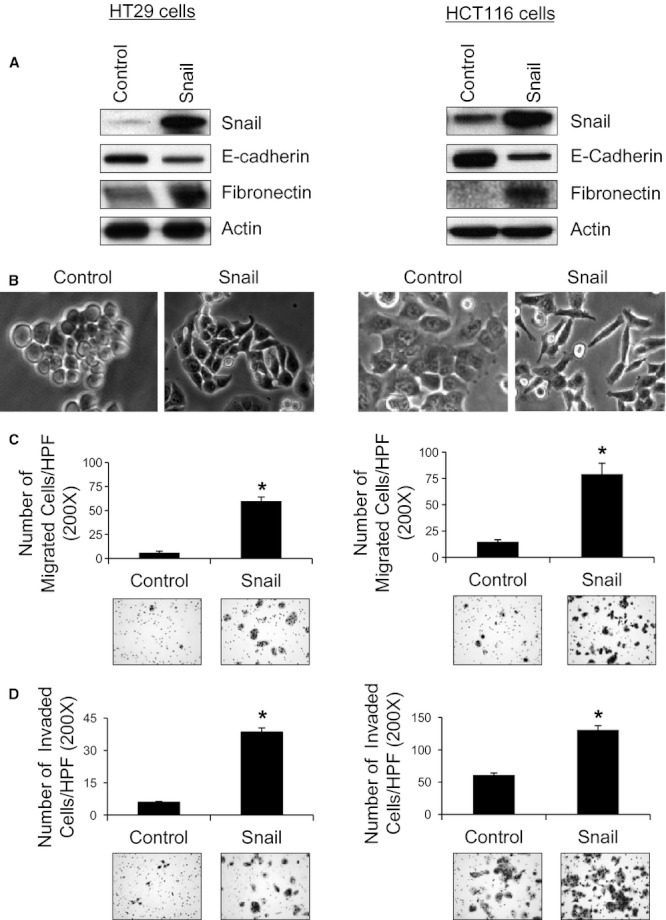
Snail overexpression in human CRC cell–lines increased cell migration and invasion. (A) Snail overexpression induced the expression of EMT markers in HT29 (left) and HCT116 (right) cells. (B) Snail overexpression led to EMT-like phenotypic changes in HT29 (left) and HCT116 (right) cells. (C) Using Boyden chamber migration assays, we found that both HT29/Snail (left) and HCT116/Snail (right) cells had increased number of migrating cells per HPF compared with control cells (*P < 0.001). (D) Using the modified Boyden chamber invasion assays, we found that HT29/Snail cells (left) had an increased number of invading cells per HPF compared with control cells (*P < 0.001). Similar results were seen in the other tested cell line, HCT116 (right, *P < 0.002).

Western blot analysis of Snail demonstrated upregulation of Snail expression in Snail-infected cells, as expected. Transfection of Snail into HT29 cells downregulated E-cadherin expression and upregulated the mesenchymal marker fibronectin (Fig. 2A, left panel). A second human CRC cell line, HCT116, was used to confirm the results obtained in HT29 cells, and it showed the same alterations in E-cadherin and fibronectin upon Snail expression (Fig. 2A, right panel). HCT116/Snail cells demonstrated increases in both vimentin and a-SMA compared to parental cells; in contrast, in HT29/Snail cells, vimentin was decreased (Fig. S1). Overexpression of Snail in HT29 (left panel) and HCT116 (right panel) cells led to a spindle-shaped morphology and loss of cell-to-cell contact (Fig. 2B). In Boyden chamber migration assays, both HT29/Snail and HCT116/Snail cells demonstrated 5-fold (HCT116) and 20-fold (HT29), increases in migration compared to their respective control cells (*P* < 0.001 for both; Fig. 2C). Similarly, in Matrigel invasion assays, both HT29/Snail and HCT116/Snail cells demonstrated increased invasion 2-fold (HCT116) and 6-fold (HT29), respectively, compared with their respective control cells (*P* < 0.002 for both; Fig. 2D).

### Overexpression of Snail induces a CSC phenotype in CRC cells

**Figure 3:**
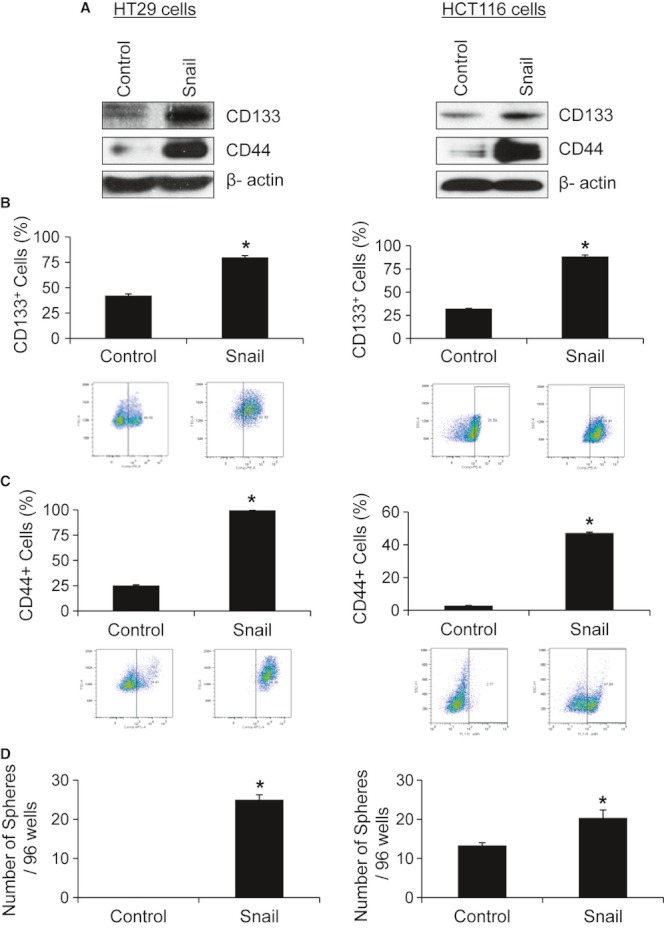
CRC cells overexpressing Snail acquire the stem cell-like phenotype. (A) HT29 cells overexpressing Snail (left) had higher expression of CD133 and CD44 protein than control cells in Western blot analysis. HCT116/Snail (right) cells demonstrated similar results. (B and C) Flow cytometry analysis was used to confirm the results obtained by Western blotting. Overexpression of Snail in HT29 (left) and HCT116 (right) had higher CD133 and CD44 expression than their respective control (*P < 0.001). (D) HT29 cells (left) overexpressing Snail demonstrated an increase in sphere formation when compared to control cells (*P < 0.001). HCT116/Snail cells (right) also formed a significantly higher number of spheres per well than control cells (*P < 0.03).

Because recent reports have shown that induction of EMT results in the acquisition of stem cell–like characteristics [11], we determined whether Snail-induced EMT could induce a stem cell–like state in CRC cells. The CSC marker profiles of CRC cells were evaluated by Western blot and flow cytometric analyses. In Western blot analysis, both HT29/Snail (left panel) and HCT116/Snail (right panel) cells expressed significantly more CD133 and CD44, putative CSC markers, than their respective controls (Fig. 3A). The number of cells that expressed CD133 in both HT29/Snail and HCT116/Snail cells were also increased 2-fold (HT29) and 3-fold (HCT116), when compared with their respective controls by flow cytometry (*P* < 0.001 for both; Fig. 3B). In HT29/Snail and HCT116/Snail cells, flow cytometry demonstrated a 4-fold (HT29) and 17-fold (HCT116) increase in CD44 expression relative to their respective controls (*P* < 0.001 for both; Fig. 3C).

CSCs can form spheres in the absence of serum under low adherence conditions. Therefore, we evaluated the ability of Snail-overexpressing cells to grow spheres under serum-free conditions. HT29/Snail cells gave rise to approximately 25-fold more spheres than HT29/control cells (*P* < 0.0001; Fig. 3D, left panel). Similarly, HCT116/Snail cells formed significantly higher numbers of spheres than control cells (*P* < 0.03; Fig. 3D, right panel).

### Overexpression of Snail mediates chemoresistance

**Figure 4:**
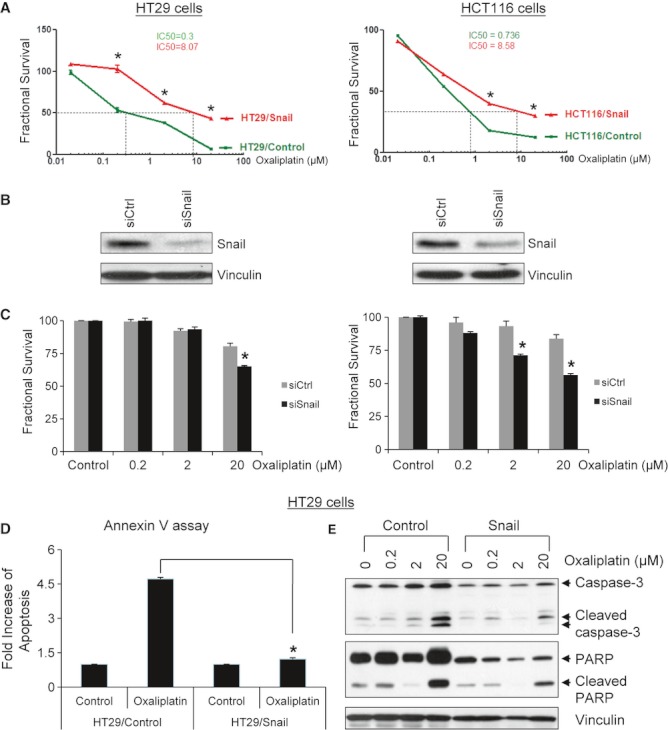
CRC cells overexpressing Snail become chemoresistant. (A) MTT assays were used to evaluate the oxaliplatin sensitivity of the Snail-overexpressing cells. Both control and Snail-transfected cells were exposed to oxaliplatin for 72 h, and the IC50 was calculated. The IC50 of HT29/Snail (left) was more than a log-fold higher than that of control cells (*P < 0.001). HCT116/Snail (right) cells had similar results (*P < 0.001). (B) Transient transfection of CRC cells with a siRNA against Snail significantly decreased expression of Snail in both HT29 (left) and HCT116 (right) cells in Western blot analysis. (C) Cell viability was significantly inhibited in both the HT29/siSnail (left) and HCT116/siSnail (right) cell lines under oxaliplatin treatment when compared to the respective control cells (*P < 0.001). (D) The number of apoptotic cells following oxaliplatin treatment was significantly lower in HT29/Snail cells than in HT29/control cells (*P < 0.001). (E) Western blot analysis revealed decreased expression of proapoptotic markers (cleaved caspase 3 and cleaved PARP) in HT29/Snail cells following oxaliplatin treatment when compared with the control cells.

Because Snail overexpression conferred a CSC-like phenotype to the CRC cells and because CSCs are hypothesized to be chemoresistant, we sought to determine the chemosensitivity of CRC cells overexpressing Snail. MTT assays demonstrated that cells overexpressing Snail exhibited greater resistance to oxaliplatin treatment than controls (Fig. 4A, left panel). The IC50 of HT29/Snail cells for oxaliplatin (8.07 μmol/L) was more than one log higher than that of control cells (0.3 μmol/L; *P* < 0.001). Similarly, the IC50 of HCT116/Snail cells for oxaliplatin (8.58 μmol/L) was more than one log higher than that of control cells (0.736 μmol/L; *P* < 0.001; Fig. 4A, right panel).

In Western blot analysis, Snail expression was downregulated in HT29 (left panel) and HCT116 (right panel) cells using transient transfection with siRNA (Fig. 4B). The effect of Snail knockdown on the chemosensitivity of HT29 and HCT116 cells was also assessed using MTT assays. HT29 and HCT116 cells using transient transfection with siRNA followed by oxaliplatin treatment for 72 h. Cell viability was significantly inhibited in both of the siRNA-transfected Snail cell lines (HT29 left panel and HCT116 right panel) compared to their respective control cells (*P* < 0.001 for both; Fig. 4C).

To further characterize the effect of Snail overexpression on chemoresistance and determine the role of apoptosis in Snail-mediated chemoresistance, Annexin V-FITC/propidium iodide double staining and flow cytometry analysis were used to assess the percentages of apoptotic cells after treatment with oxaliplatin. The percentage of apoptotic cells following oxaliplatin treatment was significantly lower in HT29/Snail cells than in HT29/control cells (*P* < 0.001; Fig. 4D), which demonstrated that the HT29/Snail cells were more chemoresistant to oxaliplatin treatment.

To confirm the role of Snail overexpression in attenuating the apoptotic response to chemotherapeutic drugs, Western blot analysis was used to assess the expression of known apoptotic markers in HT29/Snail cells following treatment with oxaliplatin. The expression of the known proapoptotic markers cleaved caspase 3 and cleaved PARP were decreased in the HT29/Snail cells when compared with the HT29/control cells (Fig. 4E).

### Overexpression of Snail in human CRC cells increases metastasis

**Figure 5:**
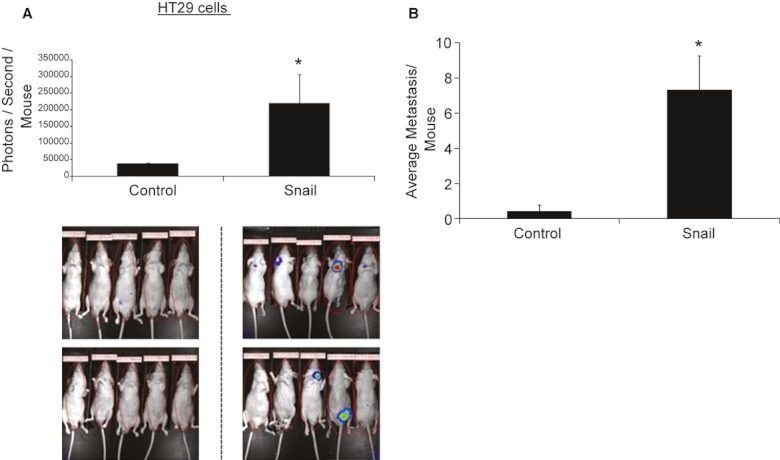
Overexpression of Snail in human CRC cells increased metastasis. (A) Luciferase-labeled HT29/control and HT29/Snail cells were injected into the tail veins of athymic nude mice. At the time of euthanasia (6 weeks and 5 days later), the group injected with HT29/Snail cells demonstrated significantly higher luciferase activity than did the control group (*P < 0.05). The bottom panel shows images of luciferase activity in all mice from each group. (B) When compared to control, overexpression of Snail in human CRC cells led to a higher average number of metastases (*P < 0.002) in mice.

Because Snail overexpression led to increased cell migration and invasion, we tested the metastatic potential of Snail-overexpressing cells in athymic nude mice. Measurement of the average bioluminescent intensity of tumor cells injected into the at the final time point (6 weeks, 5 days) showed that the group injected with HT29/Snail cells had significantly higher luciferase activity (indicating a greater tumor burden) than the control group (*P* < 0.05; Fig. 5A). Overexpression of Snail in HT29 cells led to a higher incidence of metastasis eight of the 10 mice had metastases than mice injected with control cells where only one mouse of the 10 had metastasis (*P* < 0.002). Autopsy of the mice confirmed that the group injected with HT29/Snail cells had a greater mean number of metastases than mice injected with HT29/control cells (*P* < 0.002; Fig. 5B).

## Discussion

The concepts of CSC and EMT address key aspects of tumorigenesis, growth, and metastasis. Although CSCs represent a small subset of cells within a malignant neoplasm thought to be capable of initiating the neoplasm and of driving its growth and recurrence after treatment, EMT is thought to be a critical step in the induction of tumor metastasis [1]. The relationship between EMT and CSCs has been the focus of several recent studies. Mani and colleagues [11] demonstrated that induction of EMT results in cells that have stem cell properties. By overexpressing the major EMT regulators Snail and Twist in immortalized human mammary epithelial cells (HMLEs), they induced an EMT-like state and conferred to the cells an increased ability to form mammospheres. When *SNAI1* and *TWIST1* were expressed in nontransformed HMLEs, *Her2*/*neu-*transformed HMLEs, and H-*ras*V12-transformed HMLEs, the cells underwent EMT and acquired greater mammosphere-forming ability and a CD44+/CD24− cell surface marker expression pattern [11]. Comparable findings were reported by Morel and colleagues [20], who demonstrated that CSCs can develop from HMLEs upon aberrant activation of the Ras/mitogen-activated protein kinase pathway. In further support for a link between EMT and CSCs, the study by McCoy et al. [21] demonstrated that the homeobox transcription factor Six1, when activated in bi-transgenic mice, induced tumors that underwent an EMT in adult mouse mammary gland epithelium and caused mammary gland cancer by increasing the population of cells displaying CSC markers. Conversely, Gupta et al. [22] reported that gene expression patterns of CSC-associated pathways were involved in EMT.

Snail is one of the master regulators that promotes EMT and mediates invasiveness as well as metastasis in many different types of malignant tumors [23, 24]. Upregulation of Snail and the associated transcriptional repression of E-cadherin may play a role in the progression of CRC [14, 15]. Analysis of Snail in human CRC in a prior study showed that 78% of the tumor samples examined overexpressed this protein [9]. Similar to our studies, those investigators also found that immunolocalization of Snail in human CRC revealed Snail staining in the primary colon cancer epithelium and in CRC liver metastasis, but Snail was largely undetectable in normal mucosa. Furthermore, analysis of nine commonly used CRC cell lines showed expression of Snail in varying degrees in all the cell lines.

Metastasis involves many steps including successful invasion, intravasation, survival in the circulation, extravasation, and colonization by the cancer cells. Many cancer cell types rely on the process of EMT for successful execution of these steps [25]. In the current study, microscopic examination revealed that CRC cells overexpressing Snail underwent a clear switch to the spindle morphology. These morphological changes have been shown to be driven by a number of molecular alterations, including loss or decrease of epithelial cell markers (e.g., E-cadherin and β-catenin) and increased expression of mesenchymal markers (e.g., N-cadherin, vimentin, and fibronectin; [2, 25, 26]. Consistent with those reports, our studies showed that Snail overexpression in CRC cells caused decreased E-cadherin expression and increased fibronectin expression. Furthermore, acquisition of a mesenchymal phenotype has been associated with invasive behavior [27, 28]. We found that CRC cells overexpressing Snail were not only more motile but also more invasive in vitro and more metastatic in vivo.

It was recently proposed that EMT enables cancer cells not only to disseminate but also to acquire the ability to self-renew by inducing a stem cell state [11]. In line with these observations, we observed that Snail-induced EMT conferred a stem cell–like phenotype in CRC cells. Overexpression of Snail in two CRC cell lines led to increased expression of the CSC markers CD133 and CD44 as well as increased ability to form colonospheres, which is characteristic of CSCs. In a recent study, Hwang et al. showed that Snail was expressed at high levels by CRC colonospheres, and overexpression of Snail in CRC cells induced most properties of colonosphere-forming cells, including cell dedifferentiation [29]. The same study further demonstrated that Snail regulates expression of the gene encoding interleukin-8 and other genes to induce CSC characteristic in CRC. Snail also mediates cell survival and is involved in the acquisition of stem cell–like characteristics in ovarian cancer cells [13]. Several lines of evidence suggest that both EMT and the CSC phenotype are associated with chemoresistance. Tumor cells undergoing EMT become resistant to chemotherapy, and tumor cells selected for drug resistance exhibit the EMT phenotype [25, 30]. Induction of EMT was shown to contribute to the decreased efficacy of chemotherapy in breast [31], colorectal [30], and ovarian [32] cancers. It has been suggested that resistance to chemotherapy is due to the existence of CSCs, which remain after treatment and lead to tumor relapse. The CSC population is particularly resistant to conventional therapies because of its altered expression of ATP-dependent drug efflux pumps [33], overexpression of the detoxification enzyme ALDH1 [34, 35], activation of DNA damage checkpoint responses [36], activation of Akt/protein kinase B survival pathways [37], and decreased production of reactive oxygen species [36, 38, 39]. Additionally, CRC CSCs have been shown to possess an autocrine and/or paracrine mechanism of interleukin-4 production that confers cell death resistance through the upregulation of antiapoptotic molecules [40]. In a recent study, Cammareri et al. [41] showed that Aurora-A is essential for the regeneration and chemoresistance of CRC CSCs. Our results show that Snail overexpression decreased the expression of the proapoptotic molecules caspase 3 and PARP in CRC. Overexpression of Snail in CRC cells were demonstrated increased Akt, Erk, and Src phosphorylation; furthermore, we found that overexpression of Snail in CRC cells led to an increase in the angiogenic factors VEGF-A, VEGF-C, and PlGF (Fig. S2). Accordingly, we found that Snail overexpression rendered CRC cells more chemoresistant to oxaliplatin. Although the exact molecular mechanisms mediating Snail-induced chemoresistance remain to be clarified, therapeutic inhibition of Snail may increase the efficacy of chemotherapeutic drugs toward the CRC stem cell population.

In summary, our studies have shown that Snail expression in human CRC cells can mediate EMT and the development of the CSC phenotype as well as chemoresistance. Therapeutic targeting of Snail may be a novel method to enhance the efficacy of chemotherapy and improve outcomes for patients with metastatic CRC.

## Supporting Information

**Figure S1.** Overexpression of Snail-induced EMT phenotype in CRC cells.

**Figure S2.** Overexpression of Snail-activated survival signaling pathways and increased secretion of angiogenic factors.
