# Supplementary material for: Overexpression of Snail induces epithelial–mesenchymal transition and a cancer stem cell–like phenotype in human colorectal cancer cells
Source: Cancer Med. 2012 Jun 8;1(1):5–16. doi: 10.1002/cam4.4 (PMC3544430; doi:10.1002/cam4.4)
Supplement: Supplementary file 1 [file cam40001-0005-SD1.pdf]

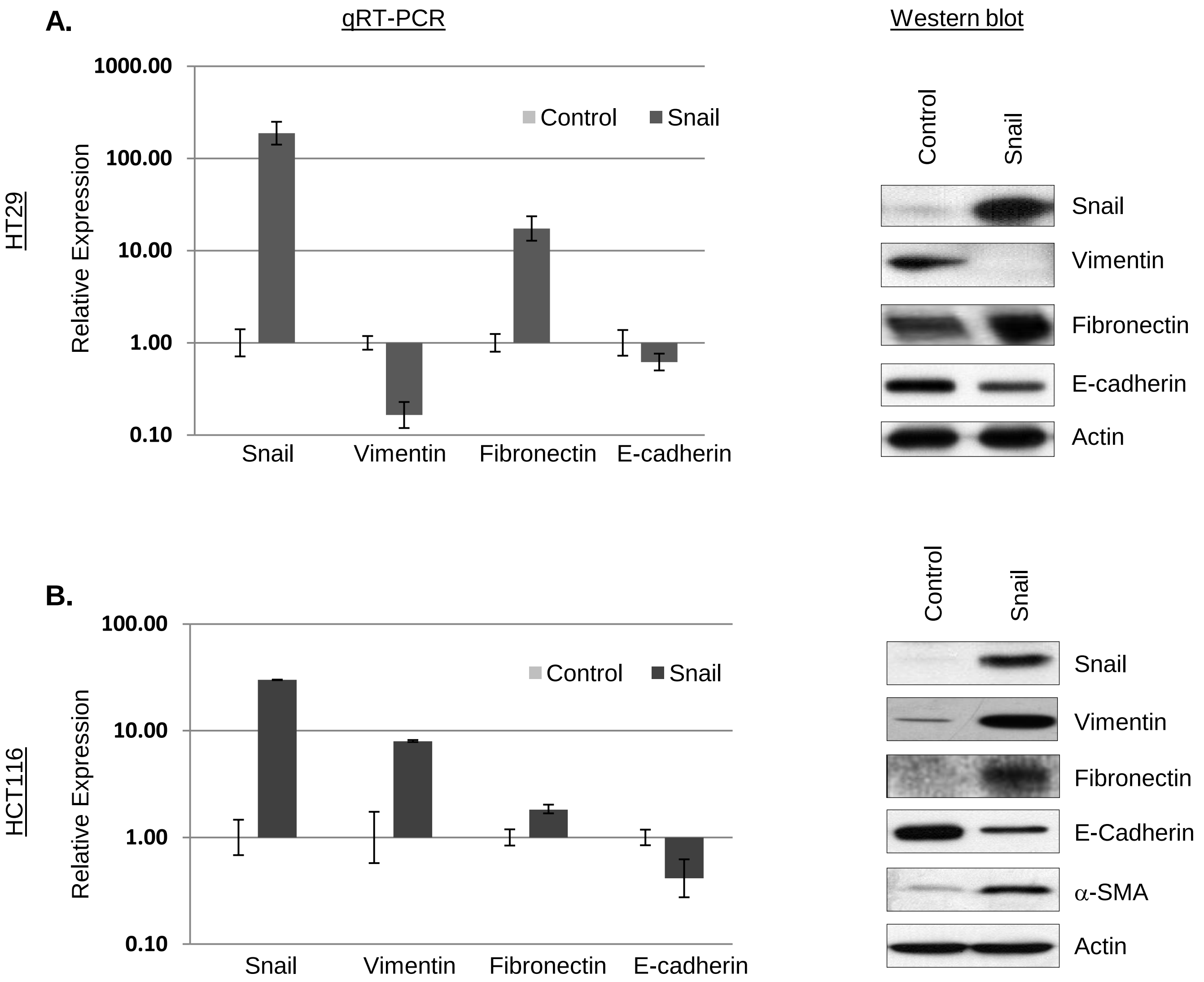

Supplementary Fig. 1. Overexpression of Snail induced EMT phenotype in CRC cells. ***A)*** Snail overexpression in HT29 cells led to changes in EMT-associated genes as demonstrated by Quantitative reverse transcription polymerase chain reaction (qRT-PCR) (panel A, left) and Western blot (panel A, right). ***B)*** A second human CRC cell line, HCT116, was used to confirm the results obtained in HT29 cells. qRT-PCR was performed in triplicate (mean ± SD).
