# Supplementary figures and images for: Overexpression of Snail induces epithelial–mesenchymal transition and a cancer stem cell–like phenotype in human colorectal cancer cells
Source: Cancer Med. 2012 Jun 8;1(1):5–16. doi: 10.1002/cam4.4 (PMC3544430; doi:10.1002/cam4.4)

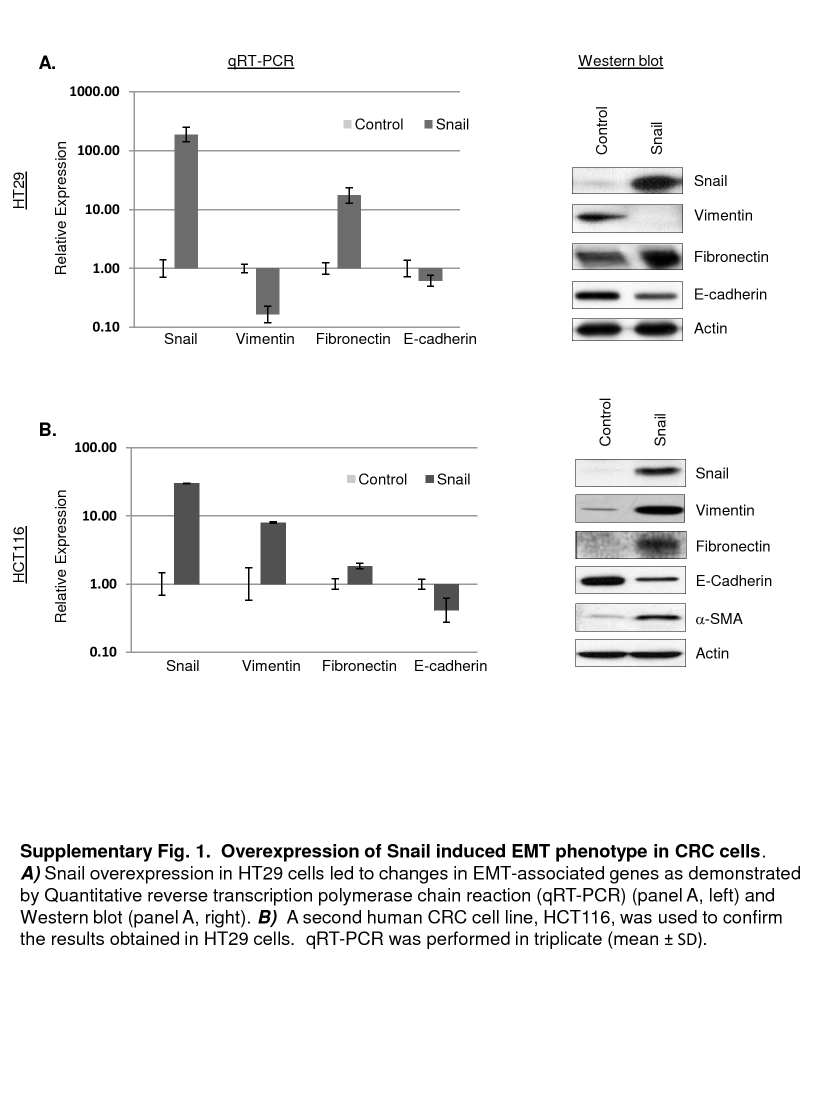

Supplement: Supplementary file 2 [file cam40001-0005-SD3.png]

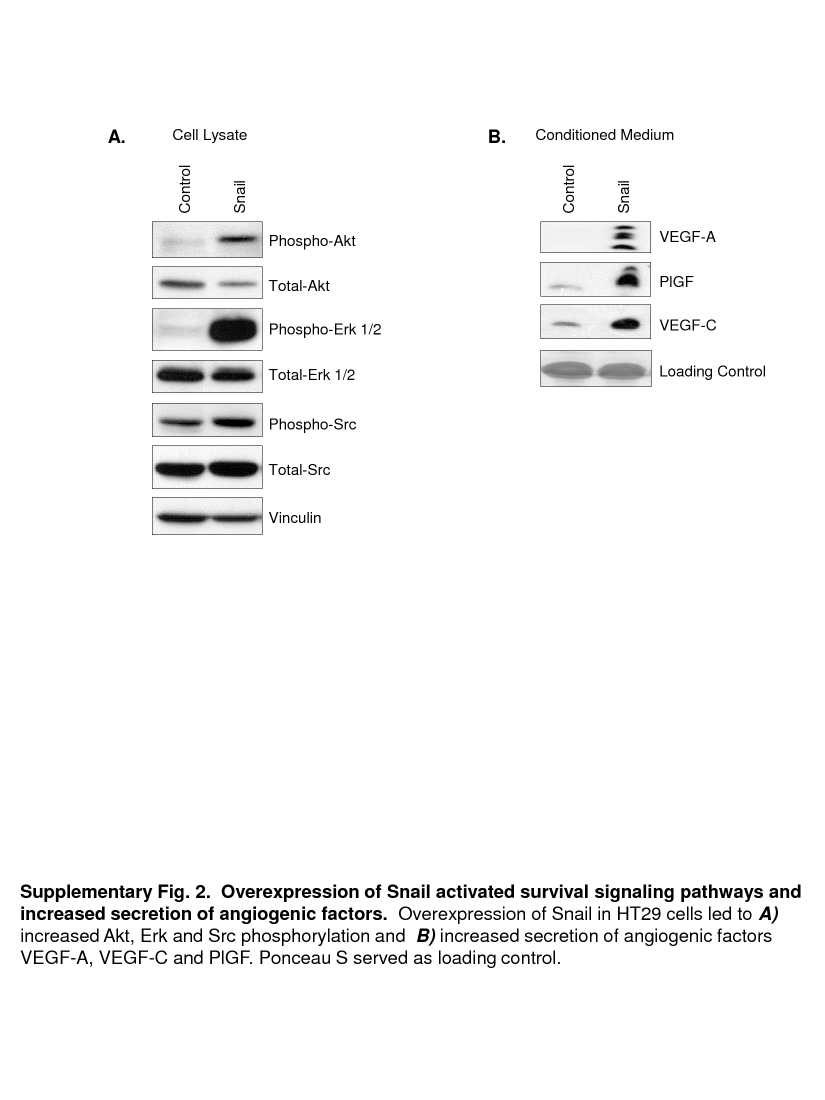

Supplement: Supplementary file 5 [file cam40001-0005-SD4.png]
