# Supplementary material for: Overexpression of Snail induces epithelial–mesenchymal transition and a cancer stem cell–like phenotype in human colorectal cancer cells
Source: Cancer Med. 2012 Jun 8;1(1):5–16. doi: 10.1002/cam4.4 (PMC3544430; doi:10.1002/cam4.4)
Supplement: Supplementary file 4 [file cam40001-0005-SD2.pdf]

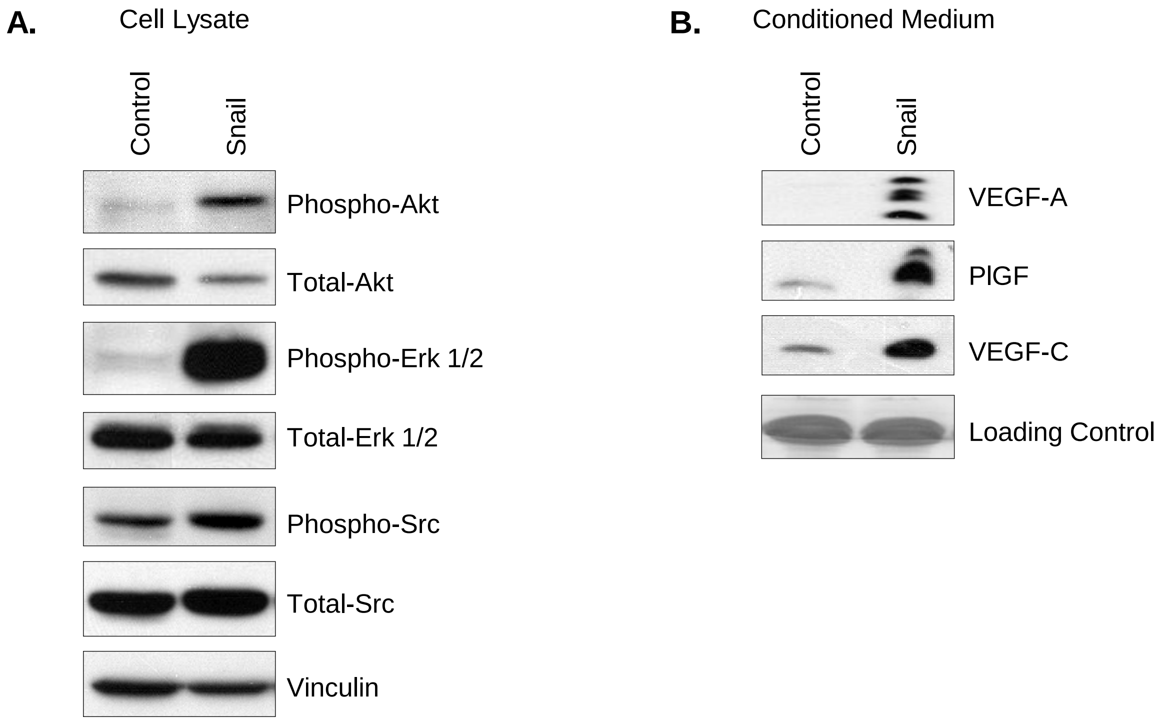


**Supplementary Fig. 2. Overexpression of Snail activated survival signaling pathways and increased secretion of angiogenic factors.** Overexpression of Snail in HT29 cells led to ***A)*** increased Akt, Erk and Src phosphorylation and ***B)*** increased secretion of angiogenic factors VEGF-A, VEGF-C and PlGF. Ponceau S served as loading control.
